# Modelling of land use land cover changes using machine learning and GIS techniques: a case study in El-Fayoum Governorate, Egypt

**DOI:** 10.1007/s10661-023-11224-7

**Published:** 2023-05-03

**Authors:** Islam Atef, Wael Ahmed, Ramadan H. Abdel-Maguid

**Affiliations:** 1 Civil Engineering Department, Faculty of Engineering, Fayoum University, Fayoum, 63514 Egypt; 2 Public Works Department, Faculty of Engineering, Cairo University, Giza, 12613 Egypt

**Keywords:** Land use land cover, GIS, Support vector machine, Change detection, Remote sensing

## Abstract

Land use/land cover (LULC) changes can occur naturally or due to human activities. In this study, the maximum likelihood algorithm (MLH) and machine learning (random forest algorithm (RF) and support vector machine (SVM)) were investigated for image classification to oversight spatio-temporal land use changes in El-Fayoum governorate, Egypt. The Google Earth Engine has been utilized to pre-process the Landsat imagery, and then upload it for classification. Each classification method was evaluated using field observations and high-resolution Google Earth imagery. LULC changes were assessed, utilizing Geographic Information System (GIS) techniques, over the last 20 years in three different periods: 2000–2012, 2012–2016, and 2016–2020. The results showed that socioeconomic changes occurred during these transitions. The SVM procedure provided the most accurate maps in terms of the kappa coefficient (0.916) compared to MLH (0.878) and RF (0.909) procedures. Therefore, the SVM technique was adopted to classify all available satellite imagery. The results of change detection showed that urban sprawl has occurred and most of the encroachments were on agricultural land. The results showed that agricultural land area decreased from 26.84% in 2000 to 26.61% in 2020 and urban area increased from 3.43% in 2000 to 5.99% in 2020. In addition, urban land expanded rapidly on account of agricultural lands by a total of 4.78% from 2012 to 2016, while it expanded slowly by a total of 3.23% from 2016 to 2020. Overall, this study offers useful insight into LULC changes that might aid shareholders and decision makers in making informed decisions.

## Introduction

Studying changes on the Earth’s surface is vital to understanding ecological and social change. Using conventional methods and aerial photography to gather information is time-consuming and not accurate enough. It is, however, possible to observe changes in satellite images based on the most recent analysis of satellite images. In recent decades, RS imagery has been increasingly utilized to detect changes in (LULC), and vegetation. Since there are extensive collections of historical data and RS imagery, investigating human activities that have influenced LULC is easy (Rogan & Chen, 2004). It is crucial to classify LULC to detect changes. LULC classification methods and techniques for extracting accurate data about LULC from remote sensing images are highly adaptable. Various factors, such as the selection of preparation tests, the heterogeneity of the study area, the sensors used, and the number of classes to be characterized, can influence the accuracy of classification techniques (Fotso Kamga et al., 2021; Hamad, 2020). Classifiers can be grouped into different categories based on the methodology and technology employed, such as supervised and unsupervised, border and non-border classification, hard and soft (ambiguous) classification, or classifications based on Pixel and sub (Wang et al., 2020). LULC classification is no longer accurate with traditional visual interpretation and mathematical statistics (Gibril et al., 2018). A supervised LULC classification involves three main components: training samples, classifiers, and supplementary datasets (Johnson, 2015). In the last decade, numerous image classification techniques have been studied at different locations. K-nearest neighbor (Abedi & Bonyad, 2015), MLH (Ali et al., 2018), SVM (Mostafa et al., 2021; Oommen et al., 2008), and RF (Wahla et al., 2022). In recent decade, there are many study compared the classifiers as (Kulkarni & Vijaya, 2021) compared between SVM and RF for land cover classification.

Google Earth Engine (GEE) is a powerful tool for manipulating large RS images for making land cover maps over vast areas. With this platform, all remotely sensed imagery can be analyzed with a web-based code editor (IDE) without downloading them to their computers. In this way, users can easily browse, select, and assess large amounts of knowledge for a large study area (Gorelick et al., 2017). Client libraries are developed using JavaScript, while code modification is handled by Python (Pimple et al., 2018). Furthermore, GEE is unique and fashionable because of its fast processing, plus easy access to Legion algorithms that makes RS tools accessible to all users, whether they are professionals or novices (Tamiminia et al., 2020). In recent years, GEE has been featured in several research publications. To process large volumes of data efficiently, Google Earth Engine uses an architecture called Map Reduce, which divides large data sets into smaller sets and distributes them across several tools in parallel. After processing the data as individual components, output datasets were compiled. Among the satellite images included in GEE are Landsat 8, MODIS, Sentinel 2, and many others, particularly Landsat time series that span nearly 40 years. GEE has been used in many sectors like economics, health, forestry, and agriculture, according to reports (Kumar & Mutanga, 2017; Tamiminia et al., 2020).

There are several topics on which they have applied these studies, including forest and vegetation studies, and LULC studies. LULC change is employed to detect distinctions between temporally separated images. When change detection techniques are combined with inaccurate classifiers, ground surface analysis is inadequate; therefore, choosing a reliable change detection approach is crucial (Li & Cheng, 2009). Land cover change can be described as the transfer from one class to another of land cover (Ren et al., 2019). LULC approach studies dynamic changes in the global environment, various researchers are interested in it (Atay Kaya & Kut Görgün, 2020; Meyfroidt et al., 2013). The spatiotemporal alteration of LULC is documented by satellite imagery. It is feasible to identify the causes and consequences of changes allied with human activities (Cardille & Foley, 2003).

Scientists use RS and spatial analysis technologies to map and identify LULC changes ( Macleod & Congalton, 1998), in rapid land cover mapping(Yan et al., 2019), in watershed mapping (Pande, 2022), in identifying urban changes (Alqahtany, 2023; Kamel, 2020), in landscape change assessment (Huang et al., 2020), in land degradation detection (Mohamed & El-Raey, 2019), in shoreline change detection (Kouhgardi et al., 2022), in studying the climate change effect on LULC change(Tariq et al., 2022) in flood monitoring (Sharifi, 2021), tracking changes to the shoreline and reduced vegetation (Mishra et al., 2021), and numerous other applications.

In recent years, the focus of much research has been on cutting-edge machine learning algorithms, such as RF, SVM, MLH, and minimum distance (MD) (Maxwell et al., 2018). These classifiers are commonly used in LULC analysis of large historical and present-day datasets. Over the last few decades, researchers have utilized various machine learning techniques in the Fayoum region to study change detection for different study areas and times, including LULC classification analysis (Allam et al., 2019; El-Zeiny & Effat, 2017; Mandanici & Bitelli, 2015; Mohamed & El-Raey, 2019). However, no studies have compared different classifiers to study LULC changes, and none have used the Google Earth Engine (GEE) platform in their study in Fayoum. In addition, fayoum witnessed changes in urban sprawl at different rate before and after 2016, which witnessed encroachments on agricultural lands changes.

To address this gap in the research, we proposed to compare three classification algorithms (SVM, MLH, and RF) using the GEE platform in ArcGIS Pro on the whole governorate. The main contribution of this paper is to analyze Fayoum’s LULC changes using the most accurate classifier from 2000 to 2020. The objectives of this research are twofold: (1) improve the LULC classification approach through machine learning; and (2) examine the LULC changes during the past two decades (2000–2020). By providing insights into the most suitable classification algorithm and detecting changes in LULC, the results of this study will be useful for future projects and decision making. The increasing availability of satellite imagery worldwide has intensified the need for accurate LULC data.

## Study area

**Fig. 1:**
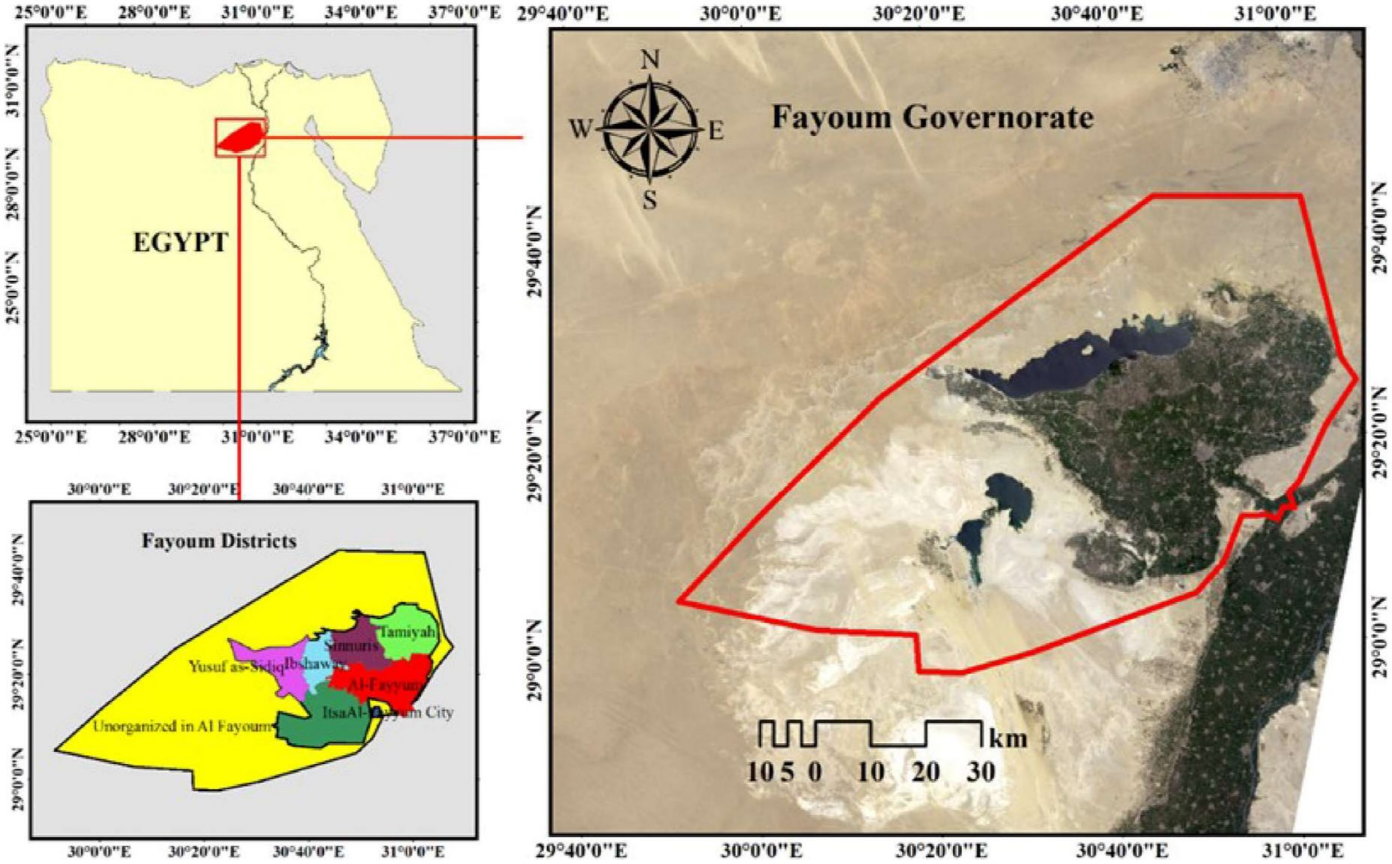
Location of El- Fayoum Governorate in Egypt

El-Fayoum is located in a noteworthy part of the depression 25 km west of the Nile, roughly 90 km southwest of Cairo, between longitudes 29° 55′ E and 31° 5 and latitudes 28° 55 N and 29° 40 N. (Fig. 1). It is located in a basin with a 1.0 m/km average land slope, sloping in a series of gardens from 26 m above mean sea level (MSL) at the Lahun regulator to 43 m below MSL at Qarun Lake. The governorate is different naturally from Upper Egypt, the delta, and the oases. In addition, rural residents and Bedouins make up the population of Fayoum. El-Fayoum depression, a beautiful oasis, is connected to the Nile by Bahr Youssef, which carries water from the El-Ibrahimiah Canal to the El-Lahoon dams, 284 km from Dariout (Farag & Donia, 2006).

The El-Fayoum region is one of the world’s oldest agricultural regions. In the midst of desert lands, it is an oasis, a lush, fertile region with abundant water. Unless the northwest depression, which fills with Qaroun Lake, the desert rises from the irrigated lands. In the Western Desert of Egypt, the Governorate occupies a relatively flat area. An area of cultivated land is estimated to be about 1,500 km2 in the El-Fayoum depression (El-Zeiny & Effat, 2017). A 2006 Egyptian census found 2,511,027 people living in the Fayoum Governorate (El-Sherbiny et al., 2016); in 2015, the total population reached 3,170,150 (Shaheen et al., 2019). The Fayoum has a hot desert climate with very hot summers and moderate winters. The average temperature in the summer is around 33 °C (91°F) and in the winter it is around 18 °C (64°F). Rainfall is scarce, with an average of only about 15 cm (6 inches) per year. The area is also subject to strong winds, especially during the winter months. The Fayoum also experiences high levels of humidity, with an average relative humidity of around 70%.

## Datasets

Landsat-8 OLI(LS8), Landsat-5(LS5), and Landsat 7 ETM + (LS) satellite images were used in this study and are freely available at GEE (https://earthengine.google.com/) and on the United States Geological Survey (USGS) website (http://glovis.usgs.gov/). We used LS5 for the year 2000, LS7 for the year 2012, and LS8 for the years (2016 and 2020). All selected imageries were selected on GEE with zero cloud cover in the specific year. The study area is covered by two scenes from Landsat (TM, ETM + , and OLI) images: 177–39 and 177–40 which merged easily on GEE. It is noteworthy that GEE will mechanically choose all pictures that run across the boundaries of the study space if the scene path is not defined, that is why we have got an oversized range of various pictures in every dataset (Phan et al., 2020). Ground truth data from Google Earth images were used to evaluate LULC classes. The two scenes of each date were assembled into a new raster image by mosaic, all corrections were made, and then the cropped image covering the area of interest was downloaded using the GEE platform.

To process images in this study, we used the computer zbook 17G3 (an Intel Core i7-6820HQ processor, an NVIDIA Quadro M3000M graphic processor, and 16 GB of RAM). The time it took for imagery to be uploaded from GEE for image processing on GEE and downloaded to use it on ARCPRO was 4 min, so GEE saved me the time of downloading the image from the US Geological Survey. Then, in Arc Pro, the processing takes 98 s (mask extraction takes 25 s, image classification takes 73 s).

## Methodology

**Fig. 2:**
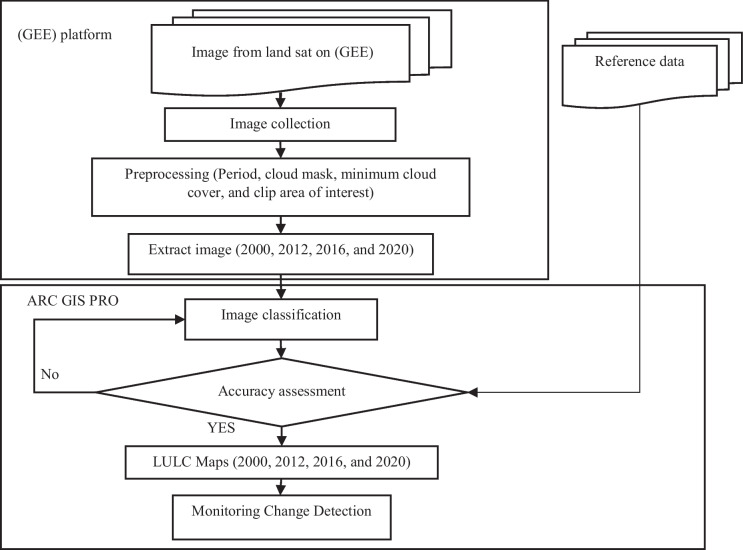
The conceptual flowchart of the applied methodology

The proposed approach, depicted in (Fig. 2), has four main steps to fulfill the aforementioned objectives: (1) application of different algorithms, (2) accuracy evaluation to select the appropriate classification algorithm, (3) LULC map generation, and (4) monitoring of the detection of changes in the space–time relationship In the forthcoming subsections will discuss the previous steps.

### Image classification

Our study employed supervised learning methods to construct a concise model of class labels using predictor features (Kotsiantis et al., 2007). Unlike unsupervised methods, this approach requires input from experts with knowledge and experience. The selection of training samples is crucial for successful model training, and in our study, all samples were manually selected using Google Earth. The RF, SVM, and MLH algorithms were applied to classify Landsat imagery for the years 2000, 2012, 2016, and 2020 into (LULC) categories: Water, Agricultural, Urban, and Desert, as outlined in Table 1. For each class, distinct training patterns were identified using visual interpretation, and the produced maps were validated using ground truth points. Based on the training samples, a signature file containing multivariate statistics of each LULC was created and used as input to the SVM, RF, and MLH classifiers in ArcGIS Pro to identify the optimal classifier. Improved accuracy and resolution of the LULC classification can be achieved by increasing the training sample size and using advanced machine learning techniques.

**Table 1:** LULC classes that exist in Fayoum governorate

No.	Class name	Description
1	Agricultural	Arable land
2	Desert	Sand and uninhabited Lands
3	Urban	Residential, road, transportation, commercial, …etc
4	Water	Waterbody

We obtained training samples specific to each year to perform a pixel-based supervised classification. In ArcGIS, we use SVM, ML, and RF classifiers.

- Random Forest (RF) is an ensemble classifier that combines multiple decision trees to improve the accuracy of the model. Developed by Breiman (2001), RF is designed to classify unknown samples using a set of trained decision trees (DTs). To select features at random, RF employs two separate processes, one for each split group. During the data splitting process, only *K* out of M features, where* K* is less than or equal to *M*, are randomly selected and used to separate the different classes. The number of trees (i.e., *P*) and the number of features at each division are the two hyper parameters that must be specified before executing the RF algorithm. In a study conducted by Shi and Yang (2016), the effect of tuning these two hyper parameters on map accuracy was examined, and it was found that different values of *P* and* K* could result in a significant difference of nine to 16% in map accuracy. The RF algorithm can be easily implemented in ArcGIS Pro, as users only need to select the number of trees in the RF interface.
- SVM is one of the supervised learning methods that is used to solve various regression and classification issues. It creates hyperplanes in spectral-radiometric features extracted using trained samples and use decision boundaries to classify different classes. Support vectors, which define the margin of the hyperplane, are the training samples utilized by SVM (Mountrakis et al., 2011). SVM incorporates kernel functions to produce kernel weights for each training sample, and the functional similarity is affected by the kernel size. To create a model using SVM, two hyper parameters are necessary: C and gamma. Gamma controls the radius of a kernel, while C controls the level of model fitting. (Mountrakis et al., 2011). While ArcGIS Pro includes an SVM classifier, there is no ability to choose C and gamma for the SVM algorithm.
- MLH is a statistical method and supervised classification technique that uses a normally distributed number to explain each band. The Byes theorem forms the foundation of this supervised classification technique (Norovsuren et al., 2019). During training, the algorithm estimates the statistical parameters of each class (mean and covariance), and then calculates the likelihood of each pixel belonging to each class based on its spectral values. The MLH classifier assigns each pixel to the class with the highest likelihood, resulting in a classified image. MLH classifier can handle multiple classes and can be used for both supervised and unsupervised classification. However, the MLH classifier may not perform well if the classes are highly overlapping or if the data has a high level of noise. Therefore, it is important to carefully select and preprocess the input data before applying the MLH classifier in ArcGIS.

### Accuracy assessment

Accuracy, precision, recall, and F1 score and* k* coefficient can also be used to evaluate the performance of an algorithm (Mohamed et al., 2022). The* K* coefficient is also often used to evaluate models in classification tasks. Image classification was validated by calculating the accuracy score derived from the error matrix to delineate whether the classification results were acceptable or unacceptable for change detection. The classified maps’ accuracy was evaluated using overall accuracy (OA), user accuracy(UA) (also referred to as recall), producer accuracy (PA) (precision), and the kappa (k) coefficient, as applied in the following researches (Jalayer et al., 2022; Shi et al., 2020).

OA is the most popular way to quantify agreement, indicating the percentage of pixels that will be correctly categorized. It can be easily calculated as shown in Eq. (1):

1 $$OA={(\textstyle\sum^{q}_{i=1}n}_{ii} /n) \times 100$$

where *q* is the total number of classes, *n* is the total number of pixels, and *n*_ii_ is the corrected classified pixels (diagonal ones).

Unfortunately, it is not possible in the existing research to obtain a clear threshold that defines the significance level for OA. Pontius and Millones (2011) indicated that the classification is accurate if the OA attains at least 85%. Karimi and Bastiaanssen (2015) consider OA suitable at 85%. As opposed to OA, UA and PA give insight into the accuracy of each category. The first, which can be distinguished as the portion of classified pixels that match the ground truth correctly, expresses, specifically, the classification’s accuracy from the user’s perspective; the accuracy of the second measure from the producer’s point of view and is determined as the percentage of ground truth pixels in the classification results that have been successfully identified (Olofsson et al., 2014). It is easy to calculate UA and PA as described in Eqs. (2) and (3):

2 $${\mathrm{UA}}_{\mathrm{i}}=({\mathrm{n}}_{\mathrm{ii}}/{\mathrm{n}}_{\mathrm{i}+})\times 100$$

3 $${\mathrm{PA}}_{\mathrm{i}}=({\mathrm{n}}_{\mathrm{ii}}/{\mathrm{n}}_{+\mathrm{i}})\times 100$$

where *n*_i+_ denotes a marginal sum of rows, and n_+i_ denotes the marginal sum of columns.

The *k* coefficient, which is expressed by Eq. (4), is utilized to measure the ratio between the actual and projected stochastic agreement if the classifier is random. The *k* coefficient is categorized as shown in (Table 2) (Brown, 2004).

**Table 2:** Kappa coefficient values range between − 1.0 and 1.0

Agreement	Kappa coefficient	
	From	To
Excellent	0.81	1.00
Good	0.61	0.80
Moderate	0.41	0.60
Weak	0.21	0.40
Bad	-1.00	0.20

4 $$k= (n\textstyle\sum_{i=1}^{r}{n}_{ii }-\textstyle\sum_{i=1}^{r}{n}_{i+}{n}_{+i} )/({n}^{2}-\textstyle\sum_{i=1}^{r}{n}_{i+}{n}_{+i})$$

Using all of these algorithms to measure accuracy allows us to model accuracy based on overall and individual class accuracy. Furthermore, comparing classifiers based only on OA and/or (k) value leads to inaccurate conclusions.

### Change detection

It is possible to extract the LULC change from various imagery datasets using a variety of techniques (Asokan & Anitha, 2019), but this procedure is not always simple or convenient. For change detection in the LULC of any area, comparing other datasets from various satellites obtained on numerous dates is a relatively simple but effective method. Some methods that utilize this methodology (Afaq & Manocha, 2021). In this study, the differences between LULC maps from the best classifier was compared to each other to ascertain the qualitative and quantitative aspects of change between the assigned periods: 2000–2012, 2012–2016, and 2016–2020. In contrast, variations in the four designated land cover classes were indicated in Table 1 for the best classifier. The maps produced from the best classifier was used to measure agricultural changes over the last two decades of the depression; urban spread over the fertile land; and the water’s surface variations.

## Results

### Image classification and accuracy assessment

**Fig. 3:**
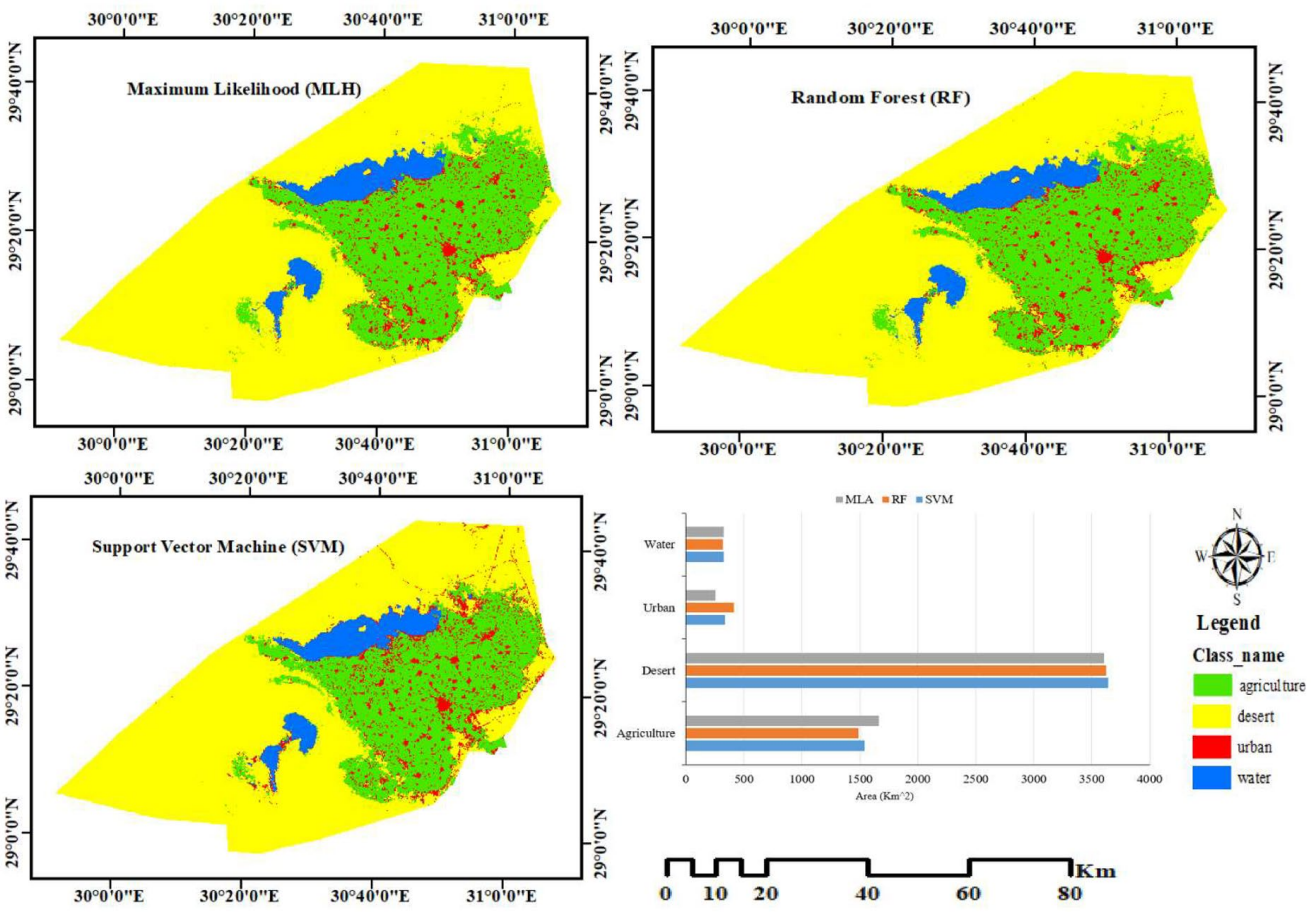
Comparison among SVM, RF, and MLH for classifying Landsat images for 2016

In this study, three supervised classification methods were evaluated on the 2016 dataset, as shown in Fig. 3 and illustrated in Table 3, and then we used a validation dataset that was distinct from the training datasets to evaluate the accuracy of each classifier. In order to assess the accuracy of these LULC classified maps in ArcGIS Pro, the OA and* k* coefficient were calculated after LULC classification using all classifiers. 370 points were tested against 370 reference samples collected from Google Earth. The total points allocated for the assessment of different land classes are 370. Out of these, 32 points have been assigned for the water class, 157 points for the agricultural class, 73 points for the urban class, and 108 points for the desert class. Table 3 shows that the SVM procedure had the highest* k* coefficient value of 0.96, compared to 0.901 and 0.92 for the MLH and RF classifiers, respectively. SVM was applied to all datasets from 2000 to 2020 for classification, and the evaluation results are presented in Table 4. It was observed that the LULC classification for the year 2000 had the lowest OA of 94.05%, while the classification for 2016 had the highest OA of 97.03%. In terms of UA and PA, the urban class had the lowest values compared to the desert class, with UA ranging from 87.50 to 94.44%, and PA ranging from 77.78 to 93.15%. The wide range of accuracy indicates that urban land is often confused with other land cover classes. The PA measure reflects the precision of the category’s prediction, while the more accurate indicator of the classification’s field utility is UA. The desert and water classes were found to be more accurate. Overall, the lowest kappa coefficient value was 0.85 for the 2000 LULC, which is still acceptable for change detection (Karimi & Bastiaanssen, 2015). SVM was utilized to create the final classified maps to monitor changes to LULC. These changes clarify how human activities, such as urbanization and encroachment on agricultural lands, affect the environment. For this assessment, four LULC categories were mapped. The governorate is mainly occupied by the desert at its northern and western borders, which is the most prevalent class in the LULC maps.

**Table 3:** Accuracy assessment of supervised classification with MLH, SVM, and RF for the 2016 dataset

Classes	**MLH**		**SVM**		**RF**	
	UA%	PA%	UA%	PA%	UA%	PA%
Agricultural	90.70	90.70	95.73	100.00	93.25	96.82
Desert	96.15	96.15	100.00	96.30	98.10	95.37
Urban	93.75	93.75	94.44	93.15	90.41	90.41
Water	100.00	100.00	100.00	93.75	100.00	90.63
OA%	93.51		97.03		94.59	
K	0.901		0.96		0.92

**Table 4:** Accuracy assessment of SVM for all datasets

**SVM**								
Classes	LULC2000		LULC2012		LULC2016		LULC2020	
	UA%	PA%	UA%	PA%	UA%	PA%	UA%	PA%
Agricultural	93.75%	90.91%	96.55%	87.50%	95.73	100.00	96.67%	90.63%
Desert	94.57%	95.60%	90.65%	97.98%	100.00	96.30	94.39%	96.19%
Urban	88.71%	90.16%	89.09%	77.78%	94.44	93.15	87.50%	87.50%
Water	99.46%	98.92%	97.21%	98.86%	100.00	93.75	97.04%	97.04%
OA%	95.95		94.05		97.03		94.60	
K	0.94		0.91		0.96		0.92	

**Fig. 4:**
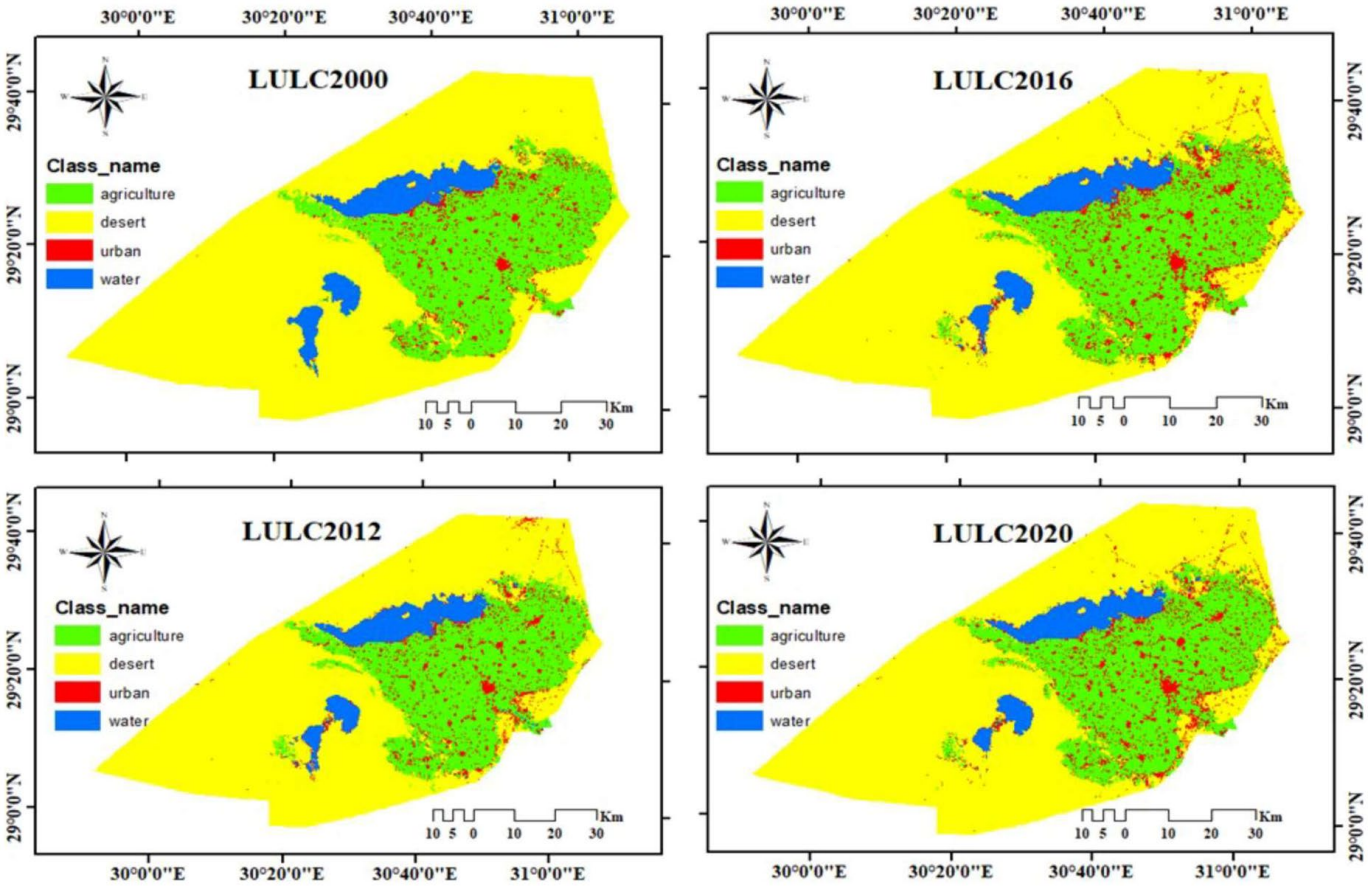
LULC maps using (SVM) algorithm for El-Fayoum governorate for four different years (2000, 2012, 2016, and 2020)

**Fig. 5:**
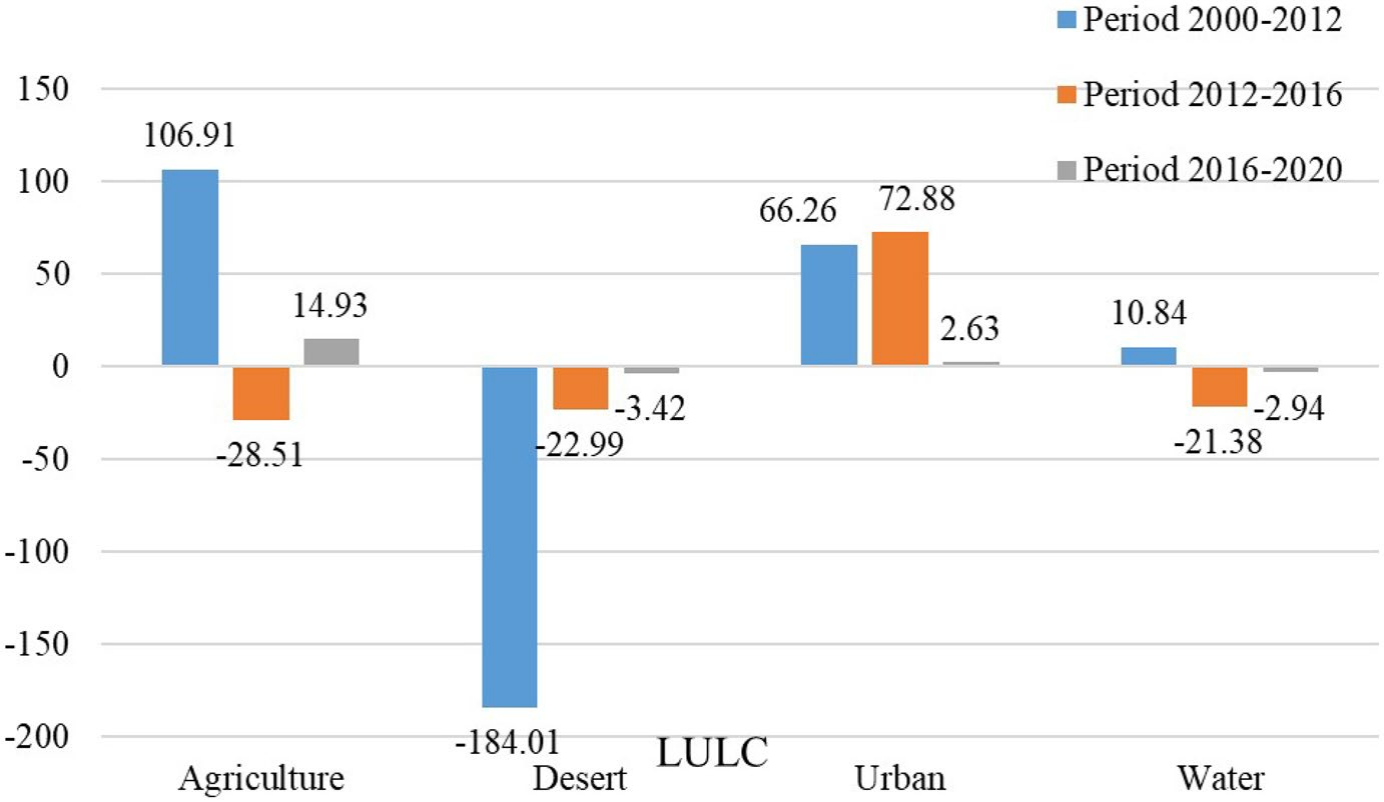
The gain and loss in (LULC) classes between 2000–2012, 2012–2016, and 2016–2020 in km^2^, in El-Fayoum, Egypt

The results of SVM show that during the chosen time as shown in Table 5 and Fig. 4, a significant change was seen in agriculture and desert classes between 2000 and 2012, But the significant change from 2012–2020 was in agriculture and urban classes. Agriculture class increased from 1463.92 km^2^ in 2000 to 1570.83 km^2^ in2012 due to land reclamation; from 2012 to 2016, it decreased from 1570.83 to 1542.32 km^2^ due to encroachment on agricultural land (urban sprawl); and finally, from 2016 to 2020, it returned to increase again from 1542.32 to 1557.24 km^2^ due to land reclamation in desert classes. The urban class increased from 200.67 km^2^ in 2000 to 350.34 km^2^ in 2020 due to the rise in population and built a new city in Fayoum in the desert area. Due to urban sprawl and land reclamation, desert classes decreased from 3849.55 km^2^ in 2000 to 3628.86 km^2^ in 2020. The water class increased from 337.73 km^2^ in 2000 to 348.57 km^2^ in 2012 due to the increase in agricultural land, which led to an increase in drainage water, then decreased from 348.57 km^2^ in 2012 to 315.42 km^2^ in 2020 due to the agriculture shrinkage. Figure 5 displays the gain and loss in LULC classes in various periods, where the agricultural class achieved the most increase and the desert class achieved the most decrease compared to other classes from 2000 to 2012.

**Table 5:** Area and percentage of each class in Fayoum Governorate (km^2^) and % of LULC classes

Classes	2000		2012		2016		2020	
	km^2^	%	km^2^	%	km^2^	%	km^2^	%
Agriculture	1463.92	25.02	1570.83	26.84	1542.32	26.36	1557.24	26.61
Desert	3849.55	65.78	3665.54	62.64	3642.55	62.25	3628.86	62.01
Urban	200.67	3.43	266.93	4.56	339.81	5.81	350.34	5.99
Water	337.73	5.77	348.57	5.96	327.19	5.59	315.42	5.39
Total	5851.87	100	5851.87	100	5851.87	100	5851.87	100

### Monitoring change detection

**Fig. 6:**
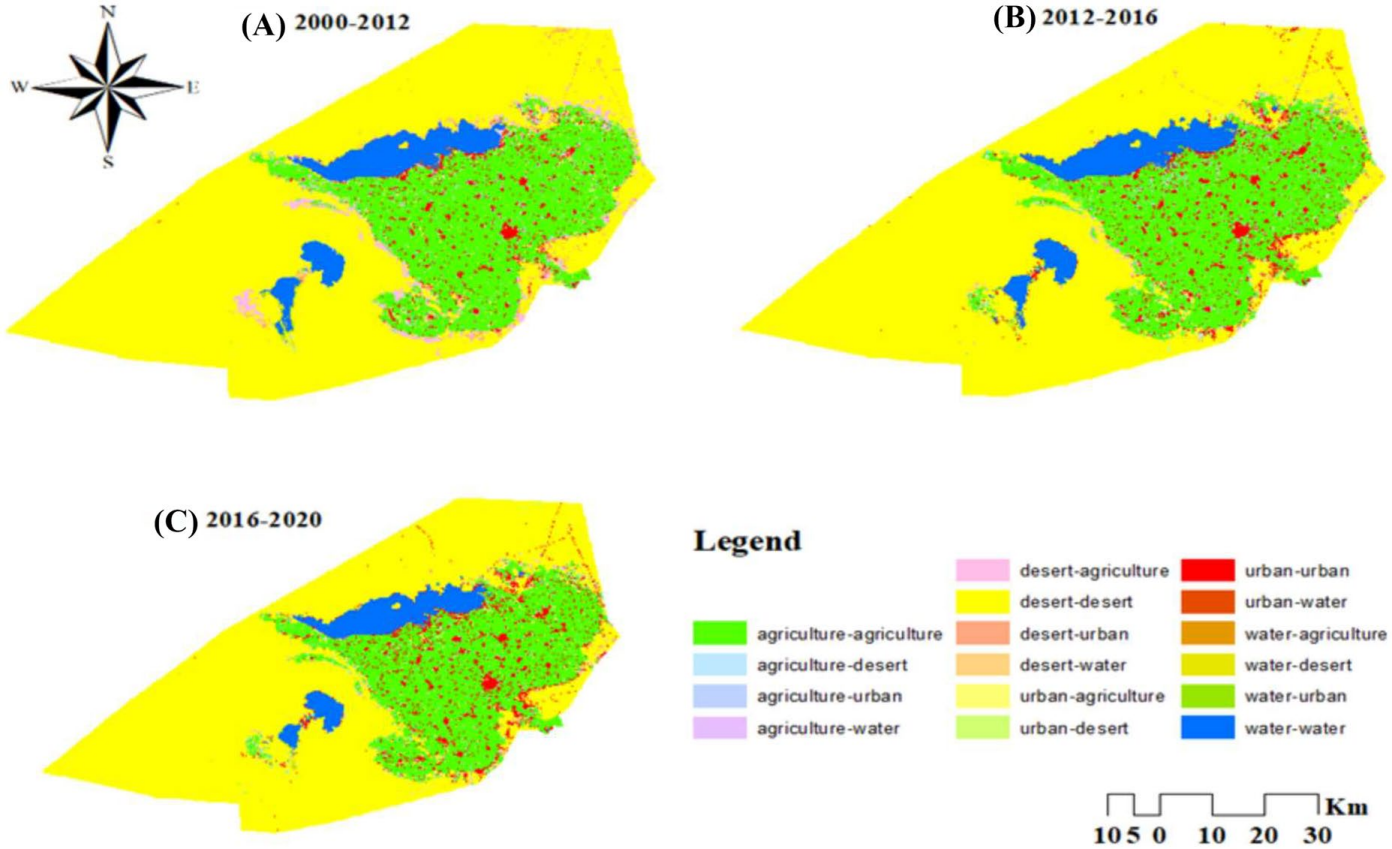
Spatial distribution of (LULC) changes between 2000–2012, 2012–2016, and 2016–2020 in EL- Fayoum, Egypt

It is easy and efficient to detect changes by comparing remotely sensed data obtained on different years. Transition matrices are also helpful in determining LULC changes. Transition matrices are useful for explaining changes in LULC classes over time. This matrix shows how much area has changed and remained the same for each LULC class (Shi et al., 2018).The transition matrix in Table 6 illustrates the change in each class over three different periods. The change detection during the first period between 2000 and 2012 indicated that ~ 95.24% (1394.3 km^2^) of the agricultural land class did not change, 0.33%(4.82 km^2^) turned to desert land, 3.85%(56.33 km^2^) turned into an urban area, and 0.58% (8.49 km^2^) turned to the water area. ~ 94.65% (3643.65 km^2^) of the desert land class was no change, while 3.23%( 124.24 km^2^) turned to agricultural land,1.81% (69.6 km^2^) to urban, and 0.31% (11.98) to water. ~ 67.65% (135.77 km^2^) of the urban area remained unchanged, while 25.64% (51.46 km^2^) turned to agricultural land, 1.54% (3.09 km^2^) to desert land, and 5.17% (10.38 km^2^) to the water area. ~ 94.07% (317.74 km^2^) of water remained unchanged, while the remaining 0.24% (0.83 km^2^), 4.13% (13.96 km^2^), and 1.55% (5.23 km^2^) were converted to agricultural land, desert land, and urban areas, respectively (Table 6 and Fig. 6). Therefore, there was a net increase in agricultural land by 106.88 km^2^, urban area by 66.24 km^2^, and water by 10.83 km^2^. But there was a net decrease in desert land by 183.95 km^2^ (Table 6 and Fig. 5).

**Table 6:** LULC change matrix among 2000–2012, 2012–2016, and 2016–2020 period’s time series is based on the SVM algorithm, In EL-Fayoum, Egypt

		LULC (2012)									
		Agriculture		Desert		Urban		Water			
		km^2^	%	km^2^	%	km^2^	%	km^2^	%	Total 2000	change class
LULC 2000	Agriculture	1394.3	95.24	4.82	0.33	56.33	3.85	8.49	0.58	1463.95	106.88
	Desert	124.24	3.23	3643.65	94.65	69.6	1.81	11.98	0.31	3849.46	-183.95
	Urban	51.46	25.64	3.09	1.54	135.77	67.65	10.38	5.17	200.7	66.24
	Water	0.83	0.24	13.96	4.13	5.23	1.55	317.74	94.07	337.76	10.83
	Total2012	1570.83		3665.51		266.94		348.59		5851.87	

		LULC (2016)									
		Agriculture		Desert		Urban		Water			
		km^2^	%	km^2^	%	km^2^	%	km^2^	%	Total 2012	change class
LULC 2012	Agriculture	1476.38	93.99	19.87	1.27	74.37	4.73	0.21	0.01	1570.83	-28.51
	Desert	28.83	0.79	3601.75	98.26	34.51	0.94	0.41	0.01	3665.5	-22.97
	Urban	29.89	11.2	17.34	6.5	218.76	81.95	0.95	0.36	266.95	72.86
	Water	7.22	2.07	3.57	1.02	12.17	3.49	325.64	93.42	348.59	-21.38
	Total2016	1542.32		3642.53		339.81		327.21		5851.87	

		LULC (2020)									
		Agriculture		Desert		Urban		Water			
		km^2^	%	km^2^	%	km^2^	%	km^2^	%	Total	change class
LULC 2016	Agriculture	1480.09	95.97%	11.2	0.73	49.89	3.23	1.13	0.07	1542.31	14.93
	Desert	26.28	0.72	3585.62	98.44	30.04	0.82	0.59	0.02	3642.53	-13.7
	Urban	47.83	14.07	25.07	7.38	265.02	77.99	1.9	0.56	339.81	10.54
	Water	3.05	0.93	6.94	2.12	5.4	1.65	311.83	95.3	327.22	-11.77
	Total	1557.25		3628.83		350.35		315.44		5851.87

From 2012 to 2016, it was found that 93.99% (1476.38 km^2^) of agricultural land did not change, while 1.27% (19.87 km^2^) turned to desert land, 4.73% (74.37 km^2^) turned into an urban area, and 0.01% (0.21 km^2^) turned to the water area. There was no change in 98.26% (3601.75 km^2^) of desert land, while 0.79% (28.83 km^2^) was transformed into agricultural land, 0.94% (34.51 km^2^) into urban, and 0.01% (0.41) to water. The urban area remained unchanged at 81.95% (218.76 km^2^), while 11.20% (29.89 km^2^) was transformed into agricultural land, 6.50% (17.34 km^2^) to desert land, and 0.36% (0.95 km^2^) to the water area. 93.42% (325.65 km^2^) of the water remained unchanged, while the remaining 2.07% (0.83 km^2^), 1.02% (3.57 km^2^), and 3.49% (12.17 km^2^) were transformed into agricultural land, desert land, and urban areas, respectively (Table 6 and Fig. 6). Therefore, there was a net increase in the urban class by 72.86 km^2^. But there was a net decrease in agricultural land by 28.51 km^2^, desert land by 22.97 km^2^, and water by 21.38 km^2^ (Table 6 and Fig. 5).

In the last period from 2016 to 2020, it was figured out that 95.97% (1480.09 km^2^) of agricultural land remained unchanged, while 0.73% (11.2 km^2^) turned to desert land, 3.23% (49.89 km^2^) turned into an urban area, and 0.07% (1.13 km^2^) turned into a water area. There was no change in 98.44% (3585.62 km^2^) of desert land, while 0.72% (26.28 km^2^) was transformed to agricultural land, 0.82% (30.04 km^2^) to urban, and 0.02% (0.59 km^2^) to water. The urban class remained unchanged at 77.99% (265.02 km^2^), while 14.07% (47.83 km^2^) was transformed into agricultural land, 7.38% (25.07 km^2^) to desert land, and 56% (1.90 km^2^) into water area 95.30% (327.22 km^2^) of water remained unchanged, while 0.93% (3.05 km^2^), 2.12% (6.94 km^2^), 1.65% (5.40 km^2^) turned to agricultural land, desert land, and urban area, respectively (Table 6, Fig. 6). Therefore, there was a net gain of 10.54 km^2^ in the urban area and 14.93 km^2^ in agricultural land. However, there was a net loss of 11.77 km^2^ of water area and 13.70 km^2^ of desert land (Table 6, Fig. 5).

## Discussion

The accuracy of classification algorithms is a crucial aspect of remote sensing analysis (Li et al., 2012), as it can directly impact the management of land use and land cover (LULC) in all classes. Several factors may drive the selection of an algorithm, including its effectiveness, ease of application, and the ability to cross-compare with earlier studies. Remote sensing analysis has been actively employed in various applications, including urban sprawl (Shao et al., 2021), agricultural lands (Goga et al., 2019), LULC change, and water resources. However, the success and relationship of these applications to actuality largely depend on the degree of accuracy attained.

In the case of Fayoum, rapid urban growth has been observed, as indicated by an increase in built area and agricultural land use (Allam et al., 2019). To discuss the benefits and drawbacks of classification algorithm studies, it is important to understand the direction of earlier studies. For instance, (El-Zeiny & Effat, 2017) utilized the (MLH) to classify Landsat images for different years, and the OA and *k* were reported. Similarly, (Allam et al., 2019) employed MLH to classify Landsat images for different years and reported the OA and k. (Mohamed & El-Raey, 2019) tested the accuracy of Landsat-7 2000 and Landsat-8 2017, and the OA was reported. However, none of these studies compared the accuracy of three machine learning algorithms. In this study, we compared among three algorithms using Landsat in 2016, and the SVM was the high accuracy in OA (97.03%) and* K* (0.96). so we used SVM to classify the all image which used monitoring the LULC changes. Overall, the choice of classification algorithm is a critical factor in remote sensing analysis, and its accuracy is vital for effective LULC management. Although various studies have employed different classification algorithms, comparing the accuracy of multiple algorithms can provide valuable insights for future research in this area.

The causative factors for LULC changes are plentiful and vary in regard to different study areas. A number of factors are responsible for changes in the LULC of Fayoum, like population expansion, encroachment on agricultural lands, and increasing unemployment rates. One of the main limitations faced in this research is that the urban land class was found to be overestimated in the generated LULC maps. This could be attributed to the fact that various waterways are so close to and adjacent to roads. As a consequence, it was difficult to fully classify and distinguish between roads and waterways in the study area using medium resolution imagery (Landsat), which led to this slight overestimation in the amount of urban land observed in the six investigated Landsat images. The results generated from this research are in accordance with a number of recent studies performed within similar areas in Fayoum to monitor the conversion between LULC. Mandanici and Bitelli (2015) stated that the agricultural areas in the Fayoum depression were 1500, 1528, and 1601 km^2^ in 1987, 2003, and 2013, respectively. According to El-Zeinya and Effatb (2017), the water class was 345.15, 342.81, 358.92, and 345.46 km^2^, the urban class was 59.13, 128.32, 217.01, and 235.94 km^2^, and the barren/desert class was 3836.67, 3765.11, 3602.42, and 3579.33 km^2^ in 1990, 2003, 2013, and 2016, respectively. According to Allam et al. (2019), the agricultural class was 1547.64, 1555.04, and 1635.15 km^2^ in 1984, 2001, and 2016, respectively, while the urban class was 43.62, 105.93, 142.40 km^2^. Their results are comparable to those that are shown here, though there may be differences in the area coverage depending on how accurately the classification was made.

## Conclusion

Through the LULC changes, human activities have altered the climate all over the world. Since the pre-industrial era, the LULC changes, such as deforestation and urbanization, have been a significant contributor to climate change (Solomon et al., 2007). So, The Egyptian authorities make great efforts to prevent unplanned urban growth on fertile land and have therefore initiated numerous projects to evaluate the spatiotemporal changes. This study used remote sensing, machine learning, and GIS technologies to monitor the spatiotemporal changes in land use and land cover (LULC) in El-Fayoum governorate. The study also proposes a new method for identifying changes in LULC using GEE for image processing and comparing the performance of multiple classifiers. Our results show that the SVM classification method achieved the highest accuracy in LULC classification as compared to RF and MLH. Human activities have significantly altered the climate through LULC changes such as desertification and urbanization, and that these changes have had a significant impact on the agricultural land in the area. The LULCC analysis and socioeconomic information have revealed a typical spatial dynamic for all fayoum. Over the past 20 years, urban or built-up areas have grown, followed by agricultural lands, which have shrunk over a specific period of time due to encroachments. Due to land reclamation and the construction of new cities there, the desert area has shrunk.

Our approach offers several advantages over previous methods, including faster and more efficient processing of large datasets, identification of the most accurate classifier for LULC change detection, and the use of GEE for LULC change detection in Fayoum, Egypt. The study also suggests future research directions, including the use of deep learning algorithms like CNN and R-CNN, to further improve the accuracy of LULC classification.

## Data Availability

All satellite images conducted in this research are free data, and LULC results during this study are included in this article.
